# Multimodality in the Collicular Pathway: Towards Compensatory Visual Processes

**DOI:** 10.3390/cells14090635

**Published:** 2025-04-25

**Authors:** Dario Rusciano, Paola Bagnoli

**Affiliations:** 1 Fidia Ophthalmic Research, 95123 Catania, Italy; drusciano55@gmail.com; 2 Department of Biology, University of Pisa, 56123 Pisa, Italy

**Keywords:** tectofugal pathway, multimodality, synaptic integration, behavioral responses

## Abstract

The integration of multisensory inputs plays a crucial role in shaping perception and behavior, particularly in the visual system. The collicular pathway, encompassing the optic tectum in non-mammalian vertebrates and the superior colliculus (SC) in mammals, is a key hub for integrating sensory information and mediating adaptive motor responses. Comparative studies across species reveal evolutionary adaptations that enhance sensory processing and contribute to compensatory mechanisms following neuronal injury. The present review outlines the structure and function of the multisensory visual pathways, emphasizing the retinocollicular projections, and their multisensory integration, which depends on synaptic convergence of afferents conveying information from different sensory modalities. The cellular mechanisms underlying multimodal integration remain to be fully clarified, and further investigations are needed to clarify the link between neuronal activity in response to multisensory stimulation and behavioral response involving motor activity. By exploring the interplay between fundamental neuroscience and translational applications, we aim to address multisensory integration as a pivotal target for its potential role in visual rehabilitation strategies.

## 1. Introduction

Among the senses, vision is perhaps the most intensely studied, and investigations of the structure and function of visual circuitries have had a predominant importance in neuroscience. Vision provides most of the information we gather from the environment. This information, once processed and interpreted, allows us to make decisions and initiate actions. The morpho-functional adaptations that evolved in different vertebrates have endowed non-mammalian species, including birds, with exceptional visual capabilities. However, studies in non-mammalian species remain few. The current reliance on rodents has led to unprecedented insights into the workings of their visual system despite their poor visual performance. Moving from the retina to the telencephalon, visual processing relies on two principal systems: the tectofugal and the thalamofugal pathway. In most mammals, visual processing mostly occurs through the thalamofugal pathway, in which retinal afferents reach the visual cortex through the dorsolateral geniculate complex. In non-mammalian vertebrates, the thalamofugal pathway often plays the lesser role, with retinal afferents reaching the forebrain through the dorsal thalamus. At variance with the thalamofugal pathway, the retino-tectal projections to the telencephalon play a major role in visuo-motor behavior, with the deep layers of the optic tectum (corresponding to the mammalian superior colliculus, SC) integrating different sensory modalities [1].

In non-mammalian vertebrates, multisensory convergence allows for integrating cross-modal inputs, leading to well-defined behavioral roles mediating detection and localization of external events and animals’ orientation to them. In mammals, multisensory integration in the SC combines cross-modal experience with influences from the association cortex, which makes unique contributions to the information-processing capabilities of the collicular neurons. When activated by different sensory modalities, the response of the SC to combined stimuli is drastically greater than its response to either of the component stimuli. Collicular efficiency in multimodal integration is particularly consolidated in humans, in which multisensory enhancement can be elicited by unconscious perception of stimuli and previous learning [2]. Knowledge of the basic principles of multisensory integration has been essential to exploit the adaptive potential of multisensory processing in a clinical setting, such as, for instance, restoring visual function in individuals with visual deficits. In this respect, the non-mammalian ability in multimodal integration, which is essential for species survival, became a strategy for visual rehabilitation in humans.

In this review, we will explore the structure and function of visual pathways, with a particular focus on retinocollicular projections and multisensory integration. Understanding multisensory processing in the SC provides key insights into adaptive mechanisms that support visual rehabilitation. When cortical damage leads to visual deficits, alternative pathways, such as the retinocollicular–extrastriate route, can compensate for impaired function. To address this, we will first examine the fundamental mechanisms of multisensory integration in the tectal/collicular pathway. We will then discuss how these mechanisms contribute to compensatory strategies in cases of visual impairment. Finally, we will explore their clinical applications and outline future directions for research and therapeutic development.

### Literature Search Methodology

We systematically searched PubMed (2010–2024) using terms like ‘superior colliculus multisensory integration, visual rehabilitation’, prioritizing peer-reviewed studies in English on non-mammals and mammals.

## 2. Conveying Visual Information to the Brain: Retinal Skill

Although the retinal circuitry is highly conserved during evolution, it has been subjected to several adjustments according to the natural environment to which the species must adapt. Major changes occurred at the level of the outer retina when vertebrates moved from water to land, where terrestrial vision required the development of novel photoreceptors responsible for color vision. Photoreceptor adjustments determined a series of changes in the inner retina, where horizontal and amacrine cells, which mediate the lateral spread of visual activation, have undergone more modifications compared to the vertical pathways that transmit information from photoreceptors to ganglion cells. Indeed, the developmental sequence—in which additional cells, both vertically and horizontally oriented, are incorporated between photoreceptors and ganglion cells—indicates greater complexity in horizontal pathways than in vertical pathways. Additionally, evolutionary divergence among cell types is more pronounced in the inner retina, suggesting that photoreception in the outer retina is highly conserved, while natural selection acts mostly on diversifying the neuronal circuitry in the inner retina that transmits information to central brain regions.

**Figure 1:**
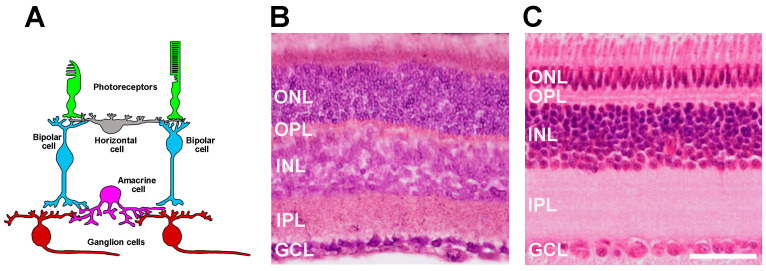
(**A**) Retinal circuitry showing the three main classes of principal neurons—photoreceptors (green), bipolar cells (light blue), and ganglion cells (red). Photoreceptors send signals to bipolar cells, which, in turn, affect the activity of ganglion cells (brown). Their axons form the optic nerve. Horizontal cells (gray) and amacrine cells (purple) modulate signal transfer across the plexiform layers. (**B**,**C**) Representative images of transversal sections from the mouse (**B**) and the chick (**C**) retina (10 µm thick, stained with hematoxylin and eosin, showing the different layers, including the outer nuclear layer (ONL), the outer plexiform layer (OPL), the inner nuclear layer (INL), the inner plexiform layer (IPL), and the ganglion cell layer (GCL) (unpublished)). The inner retina of the chick is almost twice that of the mouse, while the outer retina is thicker in the mouse with respect to the chick, presumably because of the small size of the mouse eye and the large abundance of rods in the mouse retina. Scale bar: 50 µm.

In non-mammals, the complexity of horizontal, bipolar, and ganglion cells is greater than in most mammalian species, in which the thickness of the inner retina is approximately half that of non-mammals [3]. Figure 1 shows the organization of retinal circuitry alongside representative transversal sections from the mouse and chick retina (Bagnoli, unpublished).

Beyond the specificity of cell types in providing solutions to particular behavioral challenges, the number of retinal neurons in the inner retina is a predictor of visual abilities, with an increased number of neuronal cells highly related to improved visual performance. For instance, the avian retina exhibits an increased number of displaced amacrine cells in the ganglion cell layer [4]. In this respect, the non-mammalian retina carries out functions that, in mammals, are executed by higher visual regions. Retinal skills in lower vertebrates depend on the complexity of intraretinal connections, including the number, distribution, and morphological subclasses of retinal cells, as well as their physiological responses.

A case in point is that of direction-selective circuits, which shape the output of retinal ganglion cells (RGCs) in response to specific speeds and directions of motion. Although direction selectivity was formerly attributed to the mammalian visual cortex, direction-selective responses have since been found in the retina, suggesting that visual motion analysis starts at the earliest stage of the visual hierarchy. This is even more evident at the level of the non-mammalian retina, which exhibits the most distinct sequences of visuo-motor patterns in threat avoidance and predation. For example, in chameleons, prey capture is performed through retinal image enlargement and the unique capacity to determine target distance through accommodation cues [5]. In birds, the ability to capture motion information of small objects from high altitudes requires a complex organization of the retinal network, which is further improved by midbrain neurons that efficiently extract small objects through competitive selection.

## 3. From the Optic Tectum to the Superior Colliculus

Like the optic tectum, the SC is positioned at the terminal end of the visual pathway, and it is located on the upper surface of the midbrain, where it is partially obscured by telencephalic regions, including the visual cortex in mammals.

The optic tectum in non-mammalian vertebrates and the SC in mammals share many structural and functional similarities, including a major role in multisensory integration and motor initiation. Multisensory information reaches the midbrain, where it is analyzed and filtered, enabling computational processes that determine which sensory features are relevant for adaptive behavioral responses.

In this context, the optic tectum in non-mammals and the SC in mammalian vertebrates integrate multisensory inputs with information from higher brain areas to generate a coherent motor response, allowing the animal to adapt to environmental demands (Table 1).

### 3.1. Evolutionary Conservation of Multisensory Integration

**Figure 2:**
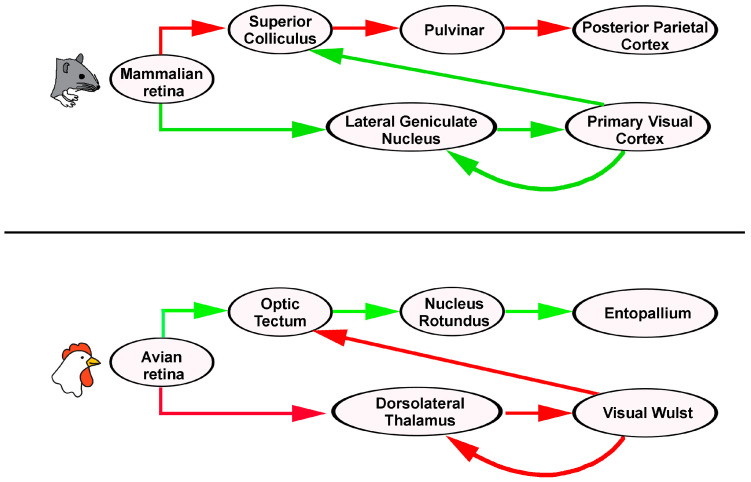
Organization of the visual system in mammals and birds. In the mouse, most ganglion cell axons project to the lateral geniculate nucleus of the dorsal thalamus from which optic radiations reach the primary visual cortex or the striate cortex (green). In the retinocollicular pathway (red), retinal projections are also directed to the extrastriate cortex via the pulvinar nucleus of the thalamus, which receives major information from the SC and plays a prominent role in sensory processing and cognitive integration. In the chick, visual information travels from the retina to the optic tectum in the midbrain, which projects to the nucleus rotundus in the thalamus, from which visual information enters the telencephalon, where it reaches the entopallium, belonging to a region considered homologous to the mammalian extrastriate cortex (green). The thalamofugal pathway (red) is homologous to the mammalian geniculo-cortical pathway, with visual information that travels from the retina to the dorsal thalamus to finally reach the visual Wulst in the telencephalon. In both mammals and birds, the striate cortex (or the visual Wulst) projects back to the thalamic and midbrain visual areas. Major visual processing in the mouse and the chick are labeled in red, while less relevant visual connections are labeled in green.

In the non-mammalian visual system, the optic tectum is part of the tectofugal pathway, which receives input from the contralateral retina and projects to the nucleus rotundus in the thalamus. The nucleus rotundus then sends projections to the ectostriatum, now called the entopallium, in the basal telencephalon [12]. The counterpart of the tectofugal pathway is the thalamofugal pathway, in which the dorsal thalamus projects to the visual Wulst, which in turn sends information to the entopallium, indicating major connections between the tectofugal and thalamofugal pathways [14]. Much research has focused on two main aspects: (1) attributing pattern discrimination to the entopallium and spatial discrimination to the Wulst, and (2) identifying homologies between non-mammalian and mammalian visual pathways. The nucleus rotundus of the thalamus shares major similarities with the pulvinar complex in mammals, while the entopallium is likely equivalent to the extrastriate cortex [21]. In Figure 2, the mouse and the chick visual systems are schematically represented, with major visual areas at the level of the midbrain, the thalamus, and the telencephalon.

### 3.2. Evolutionary Divergence and Convergence in Avian and Mammalian Visual Systems

In most avian species, a major part of the brain is devoted to the analysis of visual information, enabling birds to explore their environment for foraging and predator detection, with both converging on flight control. A fascinating recent review on avian vision [22] highlights these adaptations. From early assumptions of limited visual performance in birds compared to mammals, we now recognize advanced capabilities, such as categorical perception and concept learning, in the avian visual system [23].

The dissimilarity between avian and mammalian visual systems is striking. In primates, finely tuned visual behavior and object vision shift from subcortical centers to expansive cortical areas, although the evolutionary origins of the geniculo-cortical pathway remain debated due to deep similarities between non-mammalian and mammalian systems. Notably, the visual Wulst in birds shares functional parallels with the mammalian primary visual cortex (V1), particularly in species like the barn owl, where it exhibits orientation and binocular disparity selectivity [24]. Recent work on the lamprey further suggests that its thalamic nucleus, mediating retinal input to the pallium, may represent a prototype of the geniculate pathway [25].

These structural differences reflect divergent strategies for visually guided behavior. Unlike mammals, birds are tetrachromats, possessing four cone photoreceptor types (including ultraviolet-sensitive cones), whereas primates are trichromats, and many non-primate mammals are dichromats or monochromats. This expanded spectral range enhances color discrimination for mate selection, prey detection, and navigation [3]. They also possess a higher flicker-fusion frequency, allowing for rapid processing of dynamic visual scenes during flight, whereas mammals, adapted to slower terrestrial or subterranean movement, rely on alternative strategies [21].

Despite these differences, convergent evolution is evident. The Wulst and V1 both support orientation selectivity and binocular vision, while birds demonstrate cognitive abilities (e.g., concept learning) once attributed solely to mammals. Such parallels reveal how distinct neural architectures achieve similar perceptual outcomes, underscoring the importance of comparative studies for understanding multisensory integration in the optic tectum and the SC. In Figure 2, the organization of the visual system in mammals and birds is shown.

Research on the avian visual system has often focused on drawing homologies between non-mammalian and mammalian visual systems. Although the concept of homology is indisputably important, its pursuit might blur what truly matters in the neurophysiological strategies carried by birds to achieve excellent visual performance. Also questionable is the fact that in non-mammals, the tectofugal pathway is generally regarded as the primary route for visual information processing to the telencephalon, which is at variance with mammals, in which the retinocollicular pathway is mostly responsible for visuomotor control. It rather appears that these two pathways are capable of a diverse range of processing mechanisms, which largely overlap at different levels and with major similarities between mammalian and non-mammalian organization. Like the SC in mammals, the optic tectum in non-mammalian vertebrates is a region that integrates multisensory information, including vision and hearing, but also other sensory modalities depending on the range of natural behaviors, such as prey catching and vocal communication in birds [15]. At the structural level, the optic tectum is topographically organized, with its superficial layers receiving information from the retinal map, whereas the deep layers are multimodal and control motor activity in alignment with multisensory information.

## 4. Advantage of the Retinocollicular–Extrastriate Pathway

The visual system has a multiple-route organization, including alternative circuits, such as ganglion cell axons reaching the SC and the pulvinar, which then send information directly to higher-order visual areas (i.e., the retino-colliculo–extrastriate pathway). Additionally, projections from the lateral geniculate nucleus to extrastriate cortices bypass the primary visual cortex, offering several functional advantages. For instance, they prevent the system from complete loss of visual function in the case of damage to the geniculo-cortical pathway by ensuring visual processing at different levels [26]. In the mammalian visual system, plasticity is not limited to the geniculo-cortical pathway. Recent advances highlight how the superior colliculus’s cellular diversity and developmental connectivity underlie its capacity for experience-dependent plasticity [27]. Indeed, the retinal projections to the SC, besides being organized in a rigid manner according to fixed biochemical affinities between their axons and targets, are also inclined to some sort of flexibility, thus suggesting the possibility that the manipulation of different factors, including multisensory information underlying retinocollicular mapping, could constitute the base of a therapeutic approach to achieve regeneration after injury [28]. The SC receives topographically organized input from the eyes and the higher brain regions [29], with NMDA receptor-dependent plasticity enhancing multisensory integration [18]. The superficial layers of the SC are predominantly visual and relay information leading to visuomotor responses. A retinocollicular and a parallel corticocollicular pathway, originating from the visual cortex, converge in the superficial layers of the SC to control eye and head movements for orienting the animal with respect to visual stimulation [30]. With respect to retinocollicular projections, afferents from the cortex to the SC send information to mediate a wide range of multimodal and cognitive functions, including inputs from the prefrontal cortex that are involved in the regulation of visual attention [8]. In particular, the SC receives information from visual cortical areas and auditory cortex and somatic sensorimotor cortical areas with a topographical arrangement. In addition, the deeper layers also receive integrated information from higher-order association and prefrontal cortical areas, where spatial, auditory, visual, and somatosensory inputs are already integrated, therefore relaying integrated multisensory information to the SC. In particular, cortical projections from primary visual, auditory, and motor areas are topographically organized to allow the SC to precisely coordinate complex behaviors with respect to the sensorimotor map received by the cortical areas. Additional auditory afferents and somatosensory information to the SC are derived from the inferior colliculus, which together allow the deep collicular laminae to participate in motor response coordination. Visuomotor responses are further refined by collicular projections, reaching the lateral geniculate nucleus that projects to the primary visual cortex, from which cortical information is directed back to the SC, thus creating a loop that participates in the visuomotor response [31]. Finally, collicular output results from the integration of visual information processed in the superficial layers with multisensory information elaborated in the intermediate and deep layers, further transformed by intrinsic collicular circuits. Interactions between the superficial and the deep SC have been demonstrated using tracing techniques and electrophysiological studies. In fact, the activation of superficial collicular cells evokes monosynaptic or oligosynaptic excitatory potential in the deep SC. The fact that they are enhanced by inhibiting GABAergic transmission is indicative of intrinsic inhibitory circuits that regulate collicular output [32]. Further complexity is added by the commissural fibers arising from the tectofugal pathway directed to the contralateral SC. After the deeper layers integrate visual signals with other sensory modalities, this information is transferred to other premotor, motor, and cognitive centers to generate a behavioral action. At the behavioral level, multisensory integration increases the certainty of environmental information in an inherently noisy environment. In particular, the vast interplay between the SC and the basal ganglia facilitates the control of appropriate motor behavior, including information about the orienting motor plan. Ascending projections from the SC reach the basal ganglia via direct connections to its various nuclei or via indirect projections to thalamic and subthalamic components that influence the striatum. In particular, the SC conveys information to the limbic division of the striatum through tectonigral and tectotegmental projections, which control movements and regulate motivation. On the other hand, the striatum projects back to the receiving regions, thus providing feedback loops that exert complex control over visual information processing [16]. In fact, the basal ganglia projects back to the deep layers of the SC via the substantia nigra, which, in turn, receives input from the striatum, thus forming a closed loop from the SC to the thalamus, to the basal ganglia, and back to the SC.

**Figure 3:**
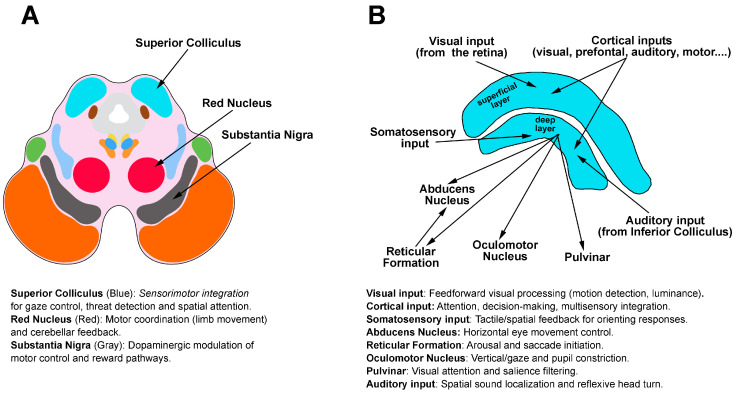
Midbrain location and SC functional organization. (**A)** Diagrammatic coronal section of the midbrain highlighting key sensorimotor hubs. (**B)** Inputs (top) and outputs (bottom) of the SC are labeled with functional roles (e.g., cortical inputs mediate attention, while abducens outputs drive eye movements). Auditory and visual streams converge here for reflexive spatial responses, with the pulvinar relaying filtered signals to higher visual areas. For clarity, functional annotations are defined in the figure.

Efferent axons from the SC are directed towards different areas, including the pulvinar of the thalamus, while additional targets in the brainstem and the spinal cord play a role in directing visual attention and regulating eye movements. In the case of visuomotor responses, visual information to the pulvinar of the thalamus provides the sensory inputs necessary for motion perception and the computation of motor responses. In addition, the efferent fibers from the SC directed towards the oculomotor nucleus and the abducens nucleus play an important role in eye movements. In this line, projections to the abducens nucleus arising from reticular formation, which receives substantial input from the SC, further support the role of the SC in visuomotor responses. To simplify the SC’s role with an example from the natural world, information from a snake approaching an individual is processed in the SC, which identifies details orienting the person away from the snake, which is perceived as a threat. In Figure 3, a diagrammatic representation of a coronal section through the midbrain at the level of the SC is shown, which is also schematically represented with its inputs and outputs.

## 5. Mechanisms of Multimodal Sensitivity

Given the central role of the SC in multisensory integration, understanding its mechanisms is crucial for evaluating the SC’s role in compensatory visual processes. In fact, the SC is pivotal in integrating visual, auditory, and somatosensory inputs, enhancing behavioral responses through multisensory plasticity, where non-visual inputs can enhance SC activity and support behavioral improvements [9]. The laminar structure of the SC leads to distinct functional properties of the different collicular layers, of which the superficial layers receive major visual input while the deep layers receive cortical input from higher-order associative areas to be coordinated with the sensorimotor networks, finally leading to SC outputs [8]. Considering that (1) almost 90% of RGCs project to the superficial layer of the SC and each SC neuron receives input from about six RGCs, (2) different types of neurons of the primary visual cortex (V1) project to the SC, and (3) the range of response properties narrows systematically with depth, reflecting an increased selectivity for behaviorally relevant visual features [13], then the SC can be seen to add functional diversity with respect to retinal output. The fact that in the deeper layers of the SC, visual information is combined with other sensory pathways, as well as motor signals, rather complicates the SC’s response properties with respect to those seen in the retina. In particular, SC multisensory neurons transform unisensory signals into a synthesized multisensory product in which physiological responses are faster, more reliable, and more robust than those elicited by each individual stimulus [10]. In non-mammals, the processing of multimodal sensory information by the optic tectum is vital for adaptive behavior in various contexts. For instance, the barn owl, an auditory specialist, is a classic model for studying multisensory integration. In the barn owl, in fact, a spatial map of auditory information is conveyed to the optic tectum for sound localization, which allows for sound-driven orienting behavior in combination with visual information acquired by the retina [6].

Multisensory integration depends on the spatial and temporal relationships between cross-modal cues, leading to synaptic convergence of afferents conveying information from different sensory modalities. However, the cellular mechanisms underlying multimodal integration remain to be fully clarified. In addition, further investigations are needed to clarify the link between neuronal activity in response to multisensory stimuli and behavioral responses involving motor neurons. At the behavioral level, the mammalian SC is involved in the processing and integration of a plethora of sensory information, ultimately influencing motor behavior. These behaviors include movements that orient the animal towards or away from external stimuli (i.e., eye movements and movements of the head and limbs) and behaviors that are organized by different brain networks but are nonetheless modulated by input from the SC, such as prey capturing and defense responses, which are crucial for animal survival. The efficacy of multisensory integration is inversely correlated with stimulus strength (inverse effectiveness), thus suggesting that integration of sensory signals would serve to enhance world perception when each unisensory signal carries some uncertainty or ambiguity. At the SC level, inverse effectiveness originates as a consequence of non-linear summation of responses to multisensory stimuli in individual collicular neurons [11]. For example, individual small synaptic inputs alone may not be sufficiently strong to recruit excitatory receptors, but paired inputs will recruit them, resulting in a response greater than the sum of those from the individual inputs [17]. The phenomenological aspects of inverse effectiveness, although well-described at the tectal/collicular level, are less known in terms of their cellular mechanisms and the generation of motor behavior. Recent advances have begun to clarify these cellular mechanisms, revealing how diverse inputs are integrated in the SC and translated into motor outputs.

Multisensory properties of collicular neurons and their integration with high-order cortical input are the subject of intense research, although their investigation is limited by the intrinsic properties of collicular neurons and the difficulty of afferent stimulation. The SC integrates different sensory modalities [through lamina-specific circuits], translating sensory information into motor commands and coordinating actions in visually guided behavior. At the cellular level, integration occurs via spatiotemporal coincidence of inputs on distinct dendrites, where NMDA-type glutamate receptor excitation interacts with intra-collicular inhibition [33]. Recent work has delineated cortico-collicular projections to specific collicular zones, each correlated with particular sensory modalities and exhibiting topographical organization [8]. Outputs are then channeled through two main pathways: (1) direct projections to brainstem motor nuclei (e.g., oculomotor circuits)] for immediate orienting responses, and (2) indirect pulvinar-extrastriate projections for perceptual integration—a mechanism that may explain residual vision in blindsight [34]. While these advances clarify how unimodal inputs are synthesized, the precise coordination between descending cortical afferents and sensorimotor networks remains less understood.

These integration principles are exemplified in the Xenopus optic tectum and the Mauthner cell system. In this respect, the optic tectum of the Xenopus, which receives synaptic input from multiple sensory modalities, provides a reliable model to study the cellular basis of multisensory integration, including inverse effectiveness, through which minor multimodal inputs overrule high-saliency unimodal information. At the cellular level, inverse effectiveness relies on non-linear response integration, which involves enhanced recruitment of local inhibition by greater unisensory stimuli compared to smaller multisensory enhancement [17]. The additional fact that repeatedly presenting a neuron with the same cross-modal (e.g., visual–auditory) stimulus enhances its sensitivity to it and to its individual stimulus has obvious translational applications for circumstances in which the efficacy of one modality has been compromised by a neurological disorder or brain injury [19,35]. This observation raises the possibility that plasticity of multisensory neurons could be involved to restore visual responsiveness when compromised. For instance, unilateral lesions of the visual cortex compromise the visual responsiveness of multisensory neurons in the ipsilateral SC to produce profound contralateral blindness (hemianopia). In this respect, multisensory plasticity at the collicular level has been found to efficiently support visual discrimination [19]. The additional fact that properties of multisensory integration are highly conserved across vertebrates, besides indicating multimodality as an important evolutionary process, also allows us to take advantage of non-mammalian models in which the simplified circuitries can make them specifically advantageous for neurosciences

Much work has been performed to investigate the synaptic mechanisms underlying multisensory integration by taking advantage of cell accessibility for in vivo intracellular recordings from neurons able to imitate complex motor behavior in response to multisensory integration. For instance, the Mauthner cells in the hindbrain of fish and amphibians are reticulospinal neurons that mediate escape behavior in response to visual and auditory stimuli. In particular, these cells can integrate visual and auditory information to increase the probability of escape behavior and to reduce response latency to weaker auditory cues, producing a relatively stronger multisensory effect [7]. In this respect, the summation of responses to low-strength stimuli from different sources enhances escape probability and reduces response latency. In contrast, the contribution to escape behavior by an intense secondary component of multisensory stimulation would make only a modest contribution to the behavioral response. At the neuronal level, afferent inputs, when activated by appropriate stimuli, give rise to excitatory postsynaptic potentials (EPSPs), which propagate down the dendrites and are summed in the cell body of the postsynaptic neuron. Subsequently, an action potential is generated when the membrane potential crosses a threshold. In the case of Mauthner cells, disynaptic auditory input arriving from the inner ear reaches the lateral dendrites, while polysynaptic visual input coming from the optic tectum contacts the ventral dendrites. Effective polysynaptic integration requires that multiple EPSPs occur almost simultaneously over a short period of time so that they overlap, leading to increased amplitude of the response after summation. In this respect, the success of synaptic integration depends on the capacitance and size of the dendritic spines and their distance from the soma, where action potentials will originate. Thus, multisensory integration depends on the geometrical properties of the postsynaptic target that determines the spatial decay for multisensory stimuli. In addition, the temporal structure of each stimulus is crucial to allow for the temporal integration of two different excitatory inputs. Whole cell recording in response to multisensory stimulation has demonstrated an enhanced response that is inversely correlated with stimulation amplitude, thus facilitating object recognition and response to sensory cues.

## 6. Compensatory Mechanisms to Recover Visual Function

Based on the seminal work of Paul Bach-y-Rita [36], substitution mechanisms can be developed as practical aids to blindness on the basis of multisensory afferents to a neural region. In the case of multisensory integration in the collicular pathway, substitution mechanisms may contribute to adaptive changes following visual impairment. Bypassing the primary visual cortex, the collicular pathway to the extrastriate cortex may underpin the remaining visual capacities associated with blindsight that is typical of patients who have been reported to show some residual visual capacity although they are considered blind [37]. For instance, after damage to V1, which is highly developed in primates, its function is partly taken by the direct pathway from the lateral geniculate nucleus to the extrastriate visual cortex or that from the SC to the pulvinar to the extrastriate cortex. Major efforts aiming to understand the mechanisms underlying visual function in blindsight patients have triggered a series of studies in animal models, demonstrating an important role of the SC–pulvinar pathway with respect to visuo-motor responses towards a target in the visual field contralateral to the lesioned V1. In addition to some contribution by the direct pathway from the lateral geniculate nucleus to the extrastriate visual cortex, a variety of cognitive functions mediated by higher brain regions can be included among additional compensatory mechanisms. Despite the limited capacity of the compensatory pathways in visual recognition, the findings from animal models have promoted in-depth understanding of the physiological recovery process, thus providing us with clues for how to treat patients with brain defects. Overall, multisensory stimulation was found to ameliorate brain deficits, such as, for instance, audio–visual training that can facilitate visual discrimination in visually impaired individuals. By leveraging cross-modal interactions, rehabilitation strategies can optimize residual neural circuits, allowing for functional recovery in patients with hemianopia or other forms of cortical blindness [19]. In patients with retinitis pigmentosa, retinal prostheses have been found to partially restore vision through cross-modal matching performance, thus implying that relearning of multisensory mappings allows for the reorganization of visual perception during blindness [38]. Additionally, there is growing evidence that targeted neurostimulation of the SC can enhance sensory integration and accelerate rehabilitation outcomes by reinforcing existing neural pathways [39]. Techniques, such as audio–visual stimulation training and sensory substitution devices capitalize on SC-mediated plasticity to improve visual function [40,41]. Additionally, emerging technologies like optogenetics and neurostimulation may offer promising avenues for reactivating SC pathways and enhancing sensory integration in patients with vision loss [42].

Multisensory-based rehabilitation leverages intact pathways like the SC–pulvinar–extrastriate route, as evidenced in blindsight patients who retain subcortical visual guidance despite V1 damage [34]. Recent optogenetic trials demonstrate that targeted SC activation enhances cross-modal integration in rodent models, as evidenced by findings that indicate successful modulation of sensory pathways in response to visual stimuli, highlighting the potential for using optogenetics to facilitate cross-modal processing in auditory and visual systems [43]. Clinical evidence suggests that auditory–visual interactions play a significant role in compensating for visual deficits, highlighting the potential for combined training to enhance spatial awareness and navigation in patients with visual loss [44]. By incorporating multisensory stimuli into training paradigms, clinicians can enhance recovery in patients with traumatic brain injuries, stroke-induced visual field deficits, and neurodegenerative conditions, including Parkinson’s disease, which is characterized by abnormal fixation and saccadic eye movements [45,46]. In autism, in addition, major impairment in drive motivation to react to emotional stimuli through face recognition might be recovered through multisensory rehabilitation approaches in the SC as a key structure in social cognitive skills [47]. Expanding the application of multisensory integration to adaptive virtual reality training and computational modeling can further refine rehabilitation methodologies and provide personalized therapeutic interventions. Although the collicular pathway is usually considered an accessory of the retino-thalamic canonical pathway, its multisensory skill gives the brain a qualitatively specific function that cannot be supplied by any other brain structure.

## 7. Multisensory Integration in the Non-Mammalian Retina

In non-mammalian vertebrates, sensory integration may begin as early as the retinal level, with RGCs displaying multisensory properties that have emerged as key players in sensory integration. The presence of multisensory processing at the level of the non-mammalian retina suggests an evolutionary advantage in pre-processing sensory information before it reaches higher brain centers. This early integration could enhance reaction times and optimize responses to environmental changes. In species with heightened reliance on multimodal perception, retinal capacity for multisensory integration might be particularly pronounced. Comparative studies have revealed that in non-mammalian species, certain RGCs receive direct mechanosensory and thermosensory inputs, reinforcing the concept that the retina serves as an active hub for sensory convergence. In amphibians and fish, retinal responses to hydrodynamic and vibrational cues suggest a broader multisensory role for retinal neurons in environmental perception [48,49]. In the zebrafish, certain RGCs have been described to integrate olfactory signals that are transmitted to the retina via the terminal nerve, the most rostral cranial nerve with chemo-sensory modality. RGC modulation was found to occur through dopamine-induced modulation of the retinal circuitry, thus providing insight into the mechanisms underlying olfacto-visual sensory integration [50,51]. These findings highlight a form of multisensory integration where olfactory information can modulate visual processing within the non-mammalian retina by facilitating early-stage sensory integration, optimizing visual processing by incorporating environmental cues and enhancing visual acuity under specific conditions. Studies in birds and reptiles further indicate that retinal integration of multimodal sensory inputs may be fundamental to behaviors like predator detection and prey tracking. For instance, avian species with high-speed flight capabilities exhibit specialized RGC types that respond to optic flow and vestibular signals simultaneously, allowing for precise navigation and obstacle avoidance. These findings suggest that the role of multisensory integration in the retina extends beyond simple light detection and is instead a crucial component of adaptive behavior [52]. The interplay between visual and non-visual inputs could enable the retina to refine incoming signals, facilitating more effective processing downstream in the visual pathway. This process would be particularly advantageous in low-light conditions or during rapid eye movements, when conventional visual processing may be challenged. In the mammalian retina, there is no evidence of RGCs capable of integrating multiple sensory modalities beyond conventional photoreception. However, some RGCs exhibit enhanced responses to visual stimuli when accompanied by mechanical stimulation, such as ocular pressure changes or vibrations transmitted through the cornea, suggesting a role in reflexive responses, such as blink reflexes or pupillary adjustments [53]. Nevertheless, the impact of multisensory integration on orienting responses, particularly in coordinating saccades with pupil responses, rather suggests the involvement of the SC due to its central role in both orienting behavior and multisensory integration [20].

In the mammalian retina, multisensory integration was presumably lost as most retinal functions shifted to higher brain regions during evolution. The retina, originally adapted for rudimentary spatial vision, evolved towards object detection and identification, enhancing its role in guidance behavior [54]. For instance, enhanced retinal function in birds may provide specific advantages to the computation that occurs in the avian telencephalon, although visual perception underwent comparable transformations in highly visual vertebrates both non-mammals and mammals [21]. On the other hand, brain complexity in mammals versus non-mammals is more intricate than expected, not only due to similarities in neural organization but also because highly sophisticated sensorimotor behaviors cannot be easily explained by simplified neuronal circuits [55].

In non-mammals, an additional strategy that might enhance retinal sensitivity depending on the location of the stimulus is the presence of centrifugal fibers, which mediate retinal control by the brain. Since the late 1980s, centrifugal fibers to the non-mammalian retina have been found to originate in a midbrain nucleus (the isthmo-optic nucleus) [56], which receives information from the optic tectum and make contacts with presumably amacrine cells in the inner nuclear layer [57]. In this respect, the classical opinion of the retina as an autonomous region that projects to the brain after processing visual stimulus fell apart despite the difficulty of obtaining information about centrifugal projections to the mammalian retina [58]. However, centrifugal afferents, although well-described in birds, still remain elusive in mammals. The recent demonstration that in the mouse retina, RGCs receive information through histaminergic neurons of the hypothalamus, which acts by improving vision of fast-moving objects, expands the notion that optomotor responses to high-speed moving stimuli might be regulated by centrifugal projections [59]. In this line, improved tracing techniques using transgenic mouse lines might help to identify additional brain regions that may also contribute to retinal processing. For instance, wide-spread projections from the locus coeruleus and the dorsal raphe would be good candidates to interact with primary sensory neurons to modulate their responsiveness to visual stimulation.

## 8. Intrinsically Photosensitive Retinal Ganglion Cells and Their Role in Complex Behavioral Responses

Intrinsically photosensitive retinal ganglion cells (ipRGCs) represent a specialized subset of retinal neurons that go beyond classical image-forming vision, playing a crucial role in both multisensory integration and compensatory plasticity in the visual system. Unlike conventional retinal ganglion cells (RGCs), which rely solely on input from rods and cones, ipRGCs possess their own light-sensitive photopigment, melanopsin, allowing them to detect ambient light levels independently [60]. However, they also integrate signals from rods and cones, effectively acting as intraretinal modulators that fine-tune retinal output based on environmental lighting conditions [60]. One key mechanism through which ipRGCs influence sensory processing is through their regulation of dopaminergic amacrine cells, which dynamically adjust retinal contrast sensitivity [61]. This modulation may help prioritize certain visual features under varying light levels—for example, enhancing motion detection in dim light to better complement auditory spatial cues, a process critical for cross-modal integration [52]. Importantly, ipRGCs do not merely relay visual information to traditional image-processing centers. Instead, their axons project to non-canonical brain regions, including the hypothalamus (for circadian rhythms and mood regulation), the olivary pretectal nucleus (for pupillary reflexes) [62,63,64], and, via indirect hypothalamic pathways, the SC, a midbrain structure essential for multisensory integration [12]. The SC is particularly significant in this context because it combines visual, auditory, and somatosensory inputs to guide orienting behaviors [11,17]. Recent research shows that hypothalamic histaminergic neurons (which receive ipRGC input) can enhance retinal responses to fast-moving stimuli [59], suggesting that ipRGCs may indirectly boost the SC’s sensitivity to dynamic audiovisual events. This aligns with the SC’s known ability to amplify weak visual signals when paired with coincident sounds or touches, a phenomenon called cross-modal enhancement [9,16].

Given their broad physiological impact, ipRGCs possess the dual ability to modulate circadian entrainment and sleep cycles in response to low-intensity light exposure while triggering acute alertness and mood regulation in response to high-intensity light stimulation [65,66]. Beyond circadian regulation, ipRGCs contribute to various reflexive behaviors, such as the photic blink reflex and light-induced avoidance responses [67]. Recent research has also explored the potential involvement of ipRGCs in mood disorders and neuropsychiatric conditions. Given their connection to higher brain regions, alterations in ipRGC function have been linked to disruptions in sleep patterns, seasonal affective disorder, and migraine photophobia [68]. In this respect, ipRGC connections to the paraventricular nucleus of the thalamus through the suprachiasmatic nucleus have been found to mediate contagious scratching behavior independently upon light detection, a finding that is indicative of ipRGC’s role in encoding stressful information [69]. The fact that ipRGCs are able to communicate back to the classical retinal circuitry suggests the possibility that they may also function to modulate the functional properties of the image-forming retinal network. This possibility, together with emerging evidence suggesting that ipRGCs can also directly contribute to non-image-forming mechanisms, suggest that they are relevant devices in compensatory visual mechanisms. In addition, the role of ipRGCs as the third class of mammalian photoreceptors and their long-lasting preservation over time highlight the potential of ipRGC-mediated function as a valuable system to eventually compensate for photoreceptor impairment. In this respect, a deeper understanding of the role of ipRGCs in circadian biology may provide insights into the mechanisms behind refractive disorders, which are likely to result from phase-shifts in circadian rhythms [70]. Even more speculative is the possibility that preserved ipRGC function might contribute to residual non-image-forming vision that may allow light-driven behavioral and physiological adjustments after visual impairment. Compensatory mechanisms at the level of ipRGCs may occur through aging to ensure the pupillary light reflex, suggesting that ipRGC’s relevance to visual disorders should not be overlooked [71].

### Compensatory Pathways in Visual Damage

Critically, ipRGCs may also support compensatory plasticity in cases of visual degeneration. In diseases like retinitis pigmentosa, where rods and cones degenerate, ipRGCs often survive longer than other RGCs [66]. Their preserved function could help maintain residual light sensitivity in the SC, even as the brain reorganizes itself to rely more on non-visual cues (e.g., auditory localization). This process involves two key compensatory mechanisms. (1) Preservation of Subcortical Light Detection: ipRGCs maintain minimal light-driven responses in the SC, allowing for rudimentary light perception despite photoreceptor loss [67]. (2) Cross-Modal Reorganization: Non-visual inputs (e.g., auditory or somatosensory) progressively “colonize” SC territories formerly dominated by vision, repurposing these circuits for alternative sensory modalities [7]. This resembles blindsight recovery, where the SC (along with pulvinar–extrastriate pathways) compensates for damage to the primary visual cortex (V1) [7]. Over time, auditory and somatosensory inputs may “colonize” SC territories that were originally vision-dominant, effectively repurposing these circuits for alternative sensory modalities. Beyond their role in sensory adaptation, ipRGCs influence higher-order functions, including circadian rhythms, mood regulation, and stress responses [60,61,62,63,64]. For instance, their connections to the paraventricular thalamus mediate light-independent behaviors (e.g., contagious scratching under stress) [64], demonstrating their broader neuromodulatory role. Additionally, their resistance to aging-related degeneration suggests they could serve as a backup system for non-image-forming vision (e.g., pupillary reflexes) even after conventional photoreceptors fail [67]. This has potential clinical relevance for (1) visual prosthetics, by harnessing ipRGC-mediated pathways to improve light-driven behaviors in blindness; (2) sensory substitution, by augmenting residual SC plasticity with auditory/tactile cues; and (3) neuroprotection through the late degeneration of ipRGCs, preserving non-image-forming functions.

However, some open questions remain that should lead to future research directions. For instance, could targeted ipRGC ablation impair the SC’s ability to compensate for cortical blindness? Or, might ipRGC-mediated pathways be harnessed for visual prosthetics or sensory substitution strategies? Finally, how do aging and disease alter ipRGC-dependent multisensory plasticity?

By bridging non-image-forming functions, multisensory integration, and compensatory mechanisms, ipRGCs emerge as a pivotal node in the brain’s adaptive response to sensory loss. Their study could unlock novel strategies for treating visual impairment and enhancing cross-modal rehabilitation.

## 9. Neural Mechanisms of Compensatory Pathway Formation

The emergence of compensatory visual pathways represents a remarkable feat of neuroplasticity, orchestrated primarily through the SC’s unique ability to integrate evolutionarily ancient survival circuits with modern multisensory processing. When damage occurs to phylogenetically newer structures like V1 or retinal networks, the SC, drawing on its deep homology with the optic tectum, activates a cascade of interdependent compensatory mechanisms that operate across multiple spatial and temporal scales. At the structural level, surviving RGC, including ipRGCs, undergo significant axonal reorganization, expanding their terminal arborizations within the SC to maintain rudimentary light detection capabilities even as conventional photoreceptors degenerate [66,67]. This anatomical remodeling occurs in tandem with competitive cross-modal invasion, where auditory and vestibular inputs progressively strengthen their synaptic footholds in SC territories formerly dominated by visual signals through activity-dependent Hebbian processes (activity-dependent plasticity mechanisms where synapses strengthen when presynaptic and postsynaptic neurons are persistently coactivated) [8,9,19,26]. The resulting structural reconfiguration effectively rewires the SC’s input landscape to prioritize preserved sensory modalities. These anatomical changes are complemented by the SC’s distinctive synaptic plasticity rules, particularly the principle of inverse effectiveness [10,17], which operates as a biological gain control system. Through this mechanism, weak residual visual signals—whether from surviving ipRGCs or intact conventional RGCs—combine with non-visual inputs to produce supralinear responses that far exceed their individual contributions. This amplification phenomenon shows striking parallels with evolutionarily conserved escape circuits in teleost fish, where combined visual and lateral line inputs generate enhanced Mauthner cell responses critical for predator avoidance [7,12,54]. At the molecular level, this process depends on precisely timed NMDA receptor activation [18,32] interacting with local inhibitory networks to create temporal windows for multisensory integration. The system-wide transition to compensatory processing is ultimately enabled by large-scale network disinhibition following cortical damage. Lesions in V1 trigger a withdrawal of GABAergic suppression across SC microcircuits [8,19,32], effectively releasing the “brakes” on subcortical inputs from the pulvinar and inferior colliculus. V1 lesions trigger SC disinhibition [19], enabling auditory/somatosensory inputs to colonize visual territories [8], a plasticity now harnessed via audio–visual training in hemianopia [40]. Translational studies have begun validating these mechanisms through two promising approaches; retinal prostheses paired with cross-modal training demonstrate significantly improved perceptual accuracy by deliberately engaging the SC’s multisensory enhancement capabilities [38], while optogenetic SC stimulation in rodent models provides direct evidence that targeted activation of these circuits can sharpen multisensory detection thresholds [42]. However, critical questions remain regarding the specific role of ipRGCs in gating SC plasticity versus their circadian functions, the biological limits of cross-modal takeover in human patients, and whether artificial induction of inverse effectiveness through neuromodulation could enhance rehabilitation outcomes, questions that bridge directly into the clinical translation strategies explored in the next section.

## 10. Optogenetic Strategies

Future research should systematically explore optogenetic strategies to enhance SC-mediated visual compensation through three complementary approaches.

First, direct modulation of SC multisensory neurons in animal models can be achieved by using improved optogenetic constructs (e.g., *ChrimsonR* for enhanced light sensitivity or *Chronos* for rapid kinetics) delivered via viral vectors to specific SC laminae. This could accurately activate multisensory integration circuits [42], potentially restoring visuomotor behaviors in cortical blindness models while allowing for detailed circuit-level analysis.

For human application, two promising pathways exist. (1) Clinical trials of retinal optogenetics (NCT03326336) demonstrate partial vision restoration by targeting surviving bipolar cells with *ChrimsonR*, bypassing degenerated photoreceptors [72]. While this approach primarily engages cortical pathways, the SC, a critical hub for visuomotor integration, remains a plausible target for future optogenetic strategies, given its role in mediating light-driven behaviors even in the absence of cortical input [42]. This approach exploits the SC’s natural plasticity and minimizes intracranial intervention risks. Recent advancements in red-shifted opsins (e.g., *bReaChES*) [73] and wearable stimulation devices are overcoming critical barriers in retinal optogenetics, including low light sensitivity thresholds and seamless integration with residual photopic vision. (2) Additionally, given that cross-modal training improves visual prosthesis outcomes [38] and TMS enhances neuroplasticity [39], future protocols could explore combining TMS with multisensory paradigms to non-invasively modulate SC function. Emerging protocols could integrate MRI-guided neuro-navigation [39] with temporally interfering electrical stimulation [74] to noninvasively target the SC. Refining these approaches may enable precise modulation of SC-mediated sensory–motor integration while ensuring safety in clinical applications.

These layered strategies would synergistically amplify residual visual signals through the retinocollicular–extrastriate pathway while integrating them with compensatory auditory and somatosensory inputs. Critical to clinical translation will be the rigorous evaluation of chronic opsin expression (particularly immune responses to viral vectors) and the optimization of stimulation parameters to avoid disrupting neural networks. Developing these approaches leverages the SC’s evolutionarily conserved role in multisensory–motor transformation, addressing practical challenges in human neurotherapeutics.

## 11. Clinical Implications and Future Directions

**Figure 4:**
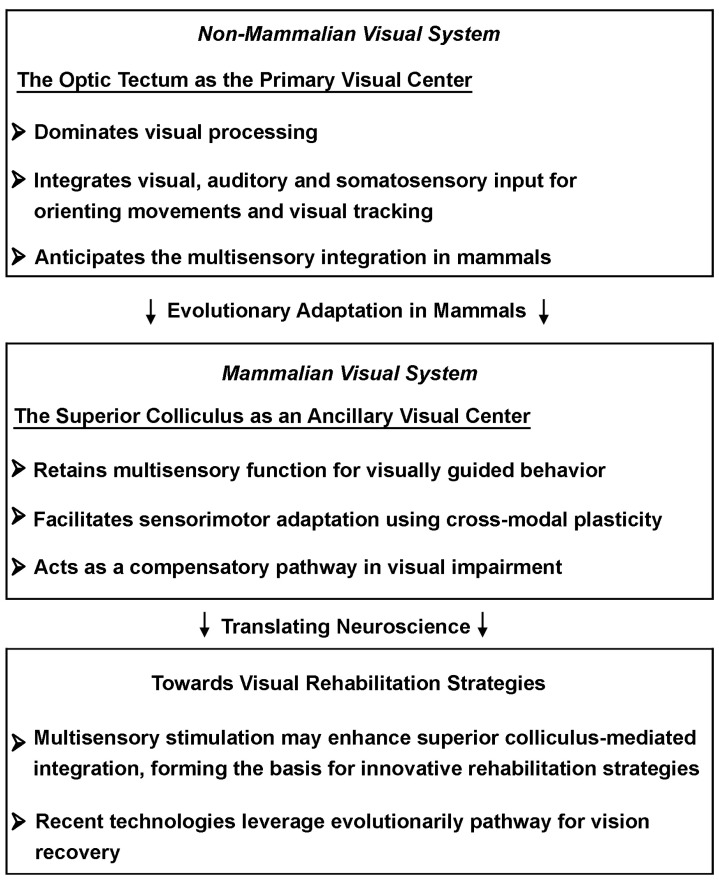
From evolutionary insights to visual rehabilitation. Diagrammatic representation of evolutionary adaptations in the optic tectum of non-mammalian vertebrates and its mammalian counterpart, the super colliculus. The transition to mammals is marked by it retaining multisensory capabilities that may possibly contribute to compensatory mechanisms following visual impairment.

Functional compensation after damage to a phylogenetically novel brain structure may occur through the reactivation of ancient systems, although their recovered performance is much lower than in the intact state. In this respect, multisensory integration in the non-mammalian optic tectum preludes the function of SC in enhancing perception and behavior across species by playing a key role in the analysis of multiple information sources. The evolutionary role of the optic tectum and its functional homology with the SC in mammals suggest that multisensory integration in subcortical pathways could be harnessed for clinical interventions in visual rehabilitation. Figure 4 summarizes how key insights from non-mammalian species translate into potential therapeutic strategies, highlighting the relevance of SC for multisensory training.

The potential of multisensory-based rehabilitation might be supported by integrating insights from basic neuroscience with translational applications. Future studies should improve our knowledge of the tectofugal visual pathway, particularly its functional organization, plasticity, and interactions with other sensory systems. At the retinal level, the contribution of ipRGCs to both image-forming and non-image-forming vision might be further clarified in order to link retinal activity to behavioral and physiological outcomes. In addition, further studies elucidating mechanisms of multimodal integration at the cellular level should be undertaken. Besides identifying the spatiotemporal coincidence of sensory information as a basic foundation for multisensory integration, much work should be performed to investigate the intracollicular circuitry underlying sensory summation and inverse effectiveness. For instance, this might include recruitment of NMDA-type glutamate receptors and intracollicular inhibitory feedback [32,33] combined with simultaneous sensory stimulation to originate enhanced responses that inversely depend on stimulation amplitude [17]. However, investigating basic mechanisms underlying multimodality at the SC level is limited by the difficulty in recording sensory activities simultaneously with their postsynaptic targets, although the advanced electrode technique has increased the potential of single-unit recordings.

In conclusion, this review presents a comprehensive overview of multisensory integration processes pointing to the evolutionary adaptation of the mammalian SC, thus offering valuable support for future investigation of basic mechanisms underlying multimodality. Whether this knowledge might be successfully translated at the rehabilitation level depends on the ability to harness the multisensory integrative properties of the SC to develop novel strategies. By leveraging SC’s capacity to process cross-modal sensory inputs, rehabilitation approaches could enhance residual visual function, improve compensatory mechanisms in visually impaired individuals, and optimize neuroplasticity-driven recovery. Future research should focus on refining multisensory training paradigms, neurostimulation techniques, and optogenetic interventions to maximize the SC’s potential in restoring functional vision.

## Figures and Tables

**Table 1:** Evolutionary comparison of multisensory integration in the optic tectum (non-mammals) and the SC (mammals), emphasizing structural and functional adaptations. See text for details.

Feature	Non-Mammalian Optic Tectum	Reference	Mammalian Superior Colliculus	Reference
Primary Sensory Inputs	Retina (direct), auditory nuclei, lateral line (fish)	[3,5,6,7]	Retina (direct + via cortex), auditory/somatosensory cortex	[8,9,10,11]
Multisensory Layers	Deep tectal laminae (stratified)	[6,12]	Intermediate/deep layers (interlaminated)	[8,13]
Motor Output Targets	Spinal cord (orienting), nucleus rotundus (thalamus)	[14,15]	Brainstem (saccades), pulvinar (cortex)	[8,16]
Plasticity Mechanisms	Spike-timing-dependent plasticity (STDP)	[7,17]	NMDA receptor-dependent LTP/LTD	[3,18]
Functional Role	Prey capture, predator avoidance	[5,7]	Visual attention, spatial navigation	[19,20]

## Data Availability

No new data were created or analyzed in this study. Data sharing is not applicable to this article.
